# Effects of rock amendment on soil physicochemical properties and organic carbon stabilization

**DOI:** 10.1016/j.isci.2025.114232

**Published:** 2025-11-26

**Authors:** Evelin Pihlap, Noemma Olagaray, Tobias Klöffel, Michael D. Masters, Rocco D'Ascanio, Ilsa B. Kantola, David J. Beerling, Noah J. Planavsky

**Affiliations:** 1 Department of Earth and Planetary Sciences, Yale University, New Haven, CT 06520-8109, USA; 2 Yale Center for Natural Carbon Capture, Yale University, New Haven, CT 06520, USA; 3 Department of Soil and Environment, Swedish University of Agricultural Sciences, 750 07 Uppsala, Sweden; 4 Department of Plant and Environmental Sciences, University of Copenhagen, 1871 Frederiksberg C, Denmark; 5 Department of Crop Sciences, University of Illinois at Urbana Champaign, Urbana, IL 61801, USA; 6 Institute for Sustainability, Energy, and Environment, Carl R. Woese Institute for Genomic Biology, University of Illinois at Urbana-Champaign, Urbana, IL 61801, USA; 7 Leverhulme Centre for Climate Change Mitigation, School of Biosciences, University of Sheffield, Sheffield S10 2TN, UK

**Keywords:** geology, soil science, soil chemistry, soil physics

## Abstract

Enhanced mineral weathering in agricultural settings is an approach for carbon dioxide removal in which crushed silicate rocks are added to soils. However, the effects of long-term application of crushed basalt on soil structure and organic carbon stabilization are still poorly constrained. We investigated a wide range of soil chemical and physical indicators in control, basalt, and lime treatment sites to provide a comprehensive evaluation of soil response to enhanced mineral weathering. The field study showed that soil organic carbon preservation was influenced by soil structure rather than rock additions. Basalt amendment improved overall soil chemical quality, and accumulating basalt from high application rates did not have any negative impact on soil physical characteristics even after six years.

## Highlights

• Basalt grains did not have any negative impact on soil physical characteristics
• Soil structural development increased from control < basalt < lime treatments
• Organo-mineral associations were unaffected by rock amendment
• Soil depth and structural characteristics influenced carbon protection

## Graphical abstract

**Figure:**
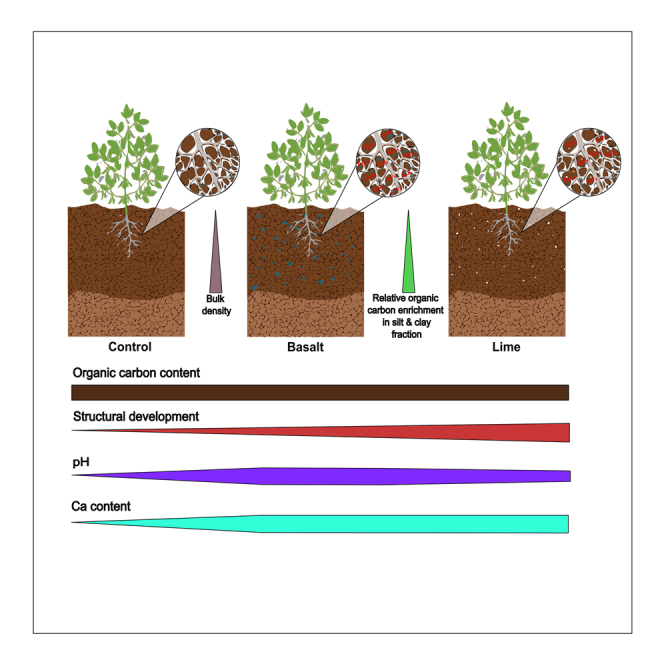

## Introduction

The effects of climate change are increasingly influencing ecosystems worldwide. For instance, agricultural ecosystems are stressed by extreme climate events (i.e., elevated temperatures, frequent droughts, and heavy rainfall), which increase concerns about crop productivity and global food security.^1^^,^^2^^,^^3^^,^^4^^,^^5^ Understanding the consequences of climate change on a global scale grows consensus that we need to not only rapidly curtail emissions, but also move to net negative emissions if we want to meet internationally agreed climate goals.^6^

Nature sequesters carbon through biotic and abiotic processes, which has inspired researchers to develop and investigate carbon dioxide removal technologies that can be implemented at a gigaton scale.^7^^,^^8^^,^^9^^,^^10^ In agricultural settings, enhanced mineral weathering (EMW) has been proposed and shown to be a promising technology for increasing carbon sequestration in soils through chemical weathering of silicate rocks.^11^^,^^12^ The core idea of EMW is to use carbonic acid present in the soil system to accelerate the chemical weathering of silicate rocks that are rich in Mg and Ca—such as basalt. The dissolution of silicate minerals by carbonic acid releases base cations and bicarbonate ions, which are transported to deeper layers of soil in solution.^13^ This process in terrestrial systems has the potential to capture billions of tons of CO_2_ annually.^14^^,^^15^^,^^16^^,^^17^

Besides carbon removal from the atmosphere, it has been hypothesized that EMW with basalt has co-benefits for crop production and soil physicochemical properties.^18^^,^^19^ Basalt in particular has been proposed to have a range of benefits over limestone addition, which is a common substrate for soil pH regulation in agricultural soils.^20^ For example, the release of cations from basalt during chemical weathering can provide nutrients and, in combination with an increase in pH, improve crop yields.^11^^,^^21^^,^^22^ Released base cations may also increase soil structural stability and carbon protection through organo-mineral associations, suggesting that EMW could be a strategy to mitigate soil organic carbon (SOC) losses,^23^^,^^24^^,^^25^ which is a topic in the EMW community that has been given less attention. Calcium cations (Ca^2+^) promote flocculation of clay particles and act as a cation bridge, linking negatively charged clay particles and organic matter (OM).^26^ Furthermore, adding fine to coarse grained basalt to soils might have the potential to provide additional charged surface area for chemical interactions^21^^,^^27^ and change soil physical properties (e.g., pore-size distribution and soil water characteristics) by altering soil grain-size distribution.^28^ It appears likely that basalt application affects numerous other soil processes such as carbon mineralization, nutrient cycling, gas diffusion, nitrification, and soil water regulation, but this has yet to be demonstrated empirically.^29^^,^^30^^,^^31^

Although the influence of basalt application on soil has been widely discussed,^19^^,^^20^^,^^21^^,^^27^^,^^32^ there are still gaps in our understanding of soil physicochemical responses because long-term field data combining soil physical and chemical characteristics are missing. Here we address this knowledge gap by investigating the change in soil physicochemical properties resulting from basalt application on a maize/soybean rotation in the corn belt region of the US (Illinois, US), which is one of the most productive agricultural regions in the world.^33^ Our goal was to test whether basalt treatment enhanced soil structure (hypothesis 1) evaluated by a new index of soil structure development (Kullback-Leibler [KL] divergence) and the resistance of aggregates to break down to finer units, which was associated with the release of divalent cations during basalt weathering. Furthermore, we tested whether additional mineral surfaces and elevated concentration of Ca^2+^ and Mg^2+^ improved the preservation of SOC due to organo-mineral interactions (hypothesis 2), thereby increasing long-term organic carbon stabilization.

## Results

### Soil chemical properties in study sites

Bulk soil chemical properties did not show any depth stratification, likely due to the homogenization from tillage. Therefore, in Table 1, we merged the data of both sampling depths. Significant differences between treatments were observed in terms of available Ca concentration, pH, and contribution of exchangeable cations (Ca^2+^ and H^+^). The pH was the highest in the basalt treatment (pH = 7.2), lower in the lime treatment (pH = 6.7), and lowest in the control treatment (pH = 6.1). In addition to soil pH, the contribution of exchangeable Ca^2+^ increased and H^+^ decreased in the basalt (80% Ca^2+^, 2% H^+^) and lime (73% Ca^2+^, 9% H^+^) treatments compared to the control site (60% Ca^2+^, 18% H^+^). This was in line with the available Ca content, where higher concentrations were in basalt treatment with 2,171 ± 178 mg Ca kg^−1^ and in lime treatment with 2,122 ± 261 mg Ca kg^−1^, whereas the content in control subplot was 1,608 ± 250 mg Ca kg^−1^. Other observed bulk soil chemical properties, such as SOC concentration, available nutrients, and CEC, were not statistically different between treatments (Table 1).

**Table 1:** Bulk soil chemical properties (both depths merged) in each treatment (± standard deviation)

Parameter	Control (*n* = 3)		Basalt (*n* = 3)		Lime (*n* = 3)	
SOC [mg g^−1^]	15	±2	15	±1	15	±2
P [mg kg^−1^]	19	±8	16	±2	21	±9
K [mg kg^−1^]	126	±19	110	±24	127	±16
Mg [mg kg^−1^]	306	±68	288	±8	317	±46
Ca [mg kg^−1^]	1608	±174^a^	2171	±151^b^	2122	±211^ab^
pH	6.1	±0.2^a^	7.2	±0.1^b^	6.7	±0.2^ab^
Buffer pH	6.8	±0.1	n.a.	n.a.	6.8	±0.1
CEC [meq 100g^−1^]	13	±2	14	±1	14	±1
Exch. K [%]	3	±1	2	±1	2	±0
Exch. Mg [%]	19	±5	18	±1	18	±2
Exch. Ca [%]	60	±2^a^	80	±1^b^	73	±4^ab^
Exch. H [%]	18	±7^a^	2	±1^b^	9	±6^ab^

The average was calculated of each field plot (*n* = 3) per treatment. Lower case letters indicate significant differences (α < 0.05) between treatments.

### Soil physical properties

The soil texture (Table 2) measured at this site was classified as silt loam (SiL; FAO), and did not differ between sampling depths and treatments. Soil water retention curves are presented in Figure S4 (1–6 cm soil depth) and S5 (15–20 cm soil depth), and the parameters of the Kosugi bimodal hydraulic are given in Table S3. The Kosugi bimodal model fitted well to the water retention measurements, where the root mean-square error (RMSE) θ remained below 0.01 throughout the dataset. Conversely, the RMSE K values for the hydraulic conductivity data were more variable, where the hydraulic conductivity curve for the basalt and lime treatments showed better fits (RMSE K ≤ 0.1) than the control (RMSE K ≥ 0.1) (Table S3). Plant-available water (W_a_), field capacity (Fc), and permanent wilting point (PWP), which were all derived from the soil water retention curve s, were in similar ranges for each sampling depth and treatment (Table 2; Figures S4 and S5). In some cases, the mean value of PWP in the basalt treatment was lower, but the change was not significant due to the high standard deviation. Soil bulk density (BD) showed depth stratification, in which lower bulk densities (1.28–1.33 g cm^−3^) were measured at 1–6 cm soil depth as compared to 15–20 cm (1.38–1.47 g cm^−3^).

**Table 2:** Soil physical properties and soil water characteristics in each treatment (± standard deviation), where BD stands for bulk density, φ for porosity, W_a_ for plant available water, Fc for field capacity at pF 1.8 and PWP for permanent wilting point at pF 4.2

Depth	Treatment	BD [g cm^−3^]	φ [-]	W_a_[Vol-%]	Fc [Vol-%]	PWP [Vol-%]	Sand [%]	Silt [%]	Clay [%]	Soil texture (FAO)
1–6 cm	Control (*n* = 3)	1.32 ± 0.08	0.50 ± 0.03	27.4 ± 3.3	35.1 ± 3.9	7.7 ± 1.1	24 ± 7	65 ± 1	11 ± 8	Silt loam (SiL)
	Basalt (*n* = 3)	1.33 ± 0.06	0.50 ± 0.03	26.7 ± 2.6	34.5 ± 3.7	7.9 ± 1.2	29 ± 11^a^	59 ± 5^a^	12 ± 6^a^	Silt loam (SiL)
	Lime (*n* = 3)	1.28 ± 0.09	0.52 ± 0.03	26.9 ± 0.9	36.2 ± 2.4	9.3 ± 1.8	14 ± 4	67 ± 4	19 ± 2	Silt loam (SiL)
15–20 cm	Control (*n* = 3)	1.47 ± 0.03	0.44 ± 0.01	24.8 ± 2.2	34.6 ± 1.6	9.8 ± 0.5	20 ± 6	67 ± 6	13 ± 1	Silt loam (SiL)
	Basalt (*n* = 3)	1.42 ± 0.03	0.46 ± 0.02	23.8 ± 2.5	32.8 ± 1.8	9.0 ± 0.8	23 ± 10^a^	62 ± 5^a^	15 ± 5^a^	Silt loam (SiL)
	Lime (*n* = 3)	1.38 ± 0.03^a^	0.48 ± 0.01^a^	24.6 ± 0.1^a^	34.5 ± 1.8^a^	9.9 ± 1.7^a^	15 ± 5	64 ± 6	21 ± 3	Silt loam (SiL)

a One field replicate was lost due to unexpected technical failures.

### Soil aggregation

**Figure 1:**
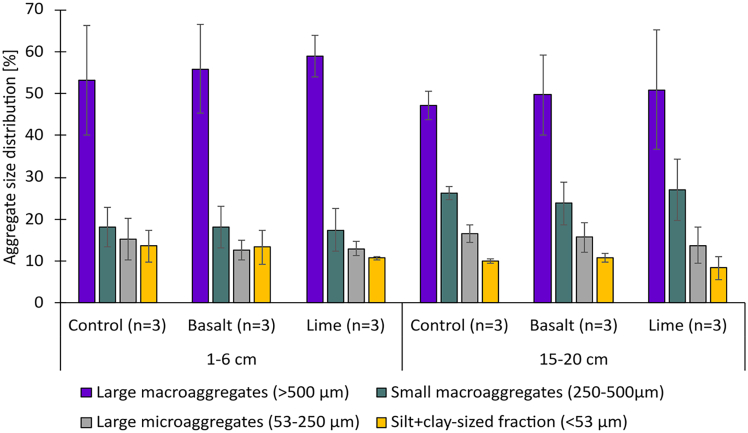
Aggregate size class distribution at the 1–6 cm and 15–20 cm

**Figure 2:**
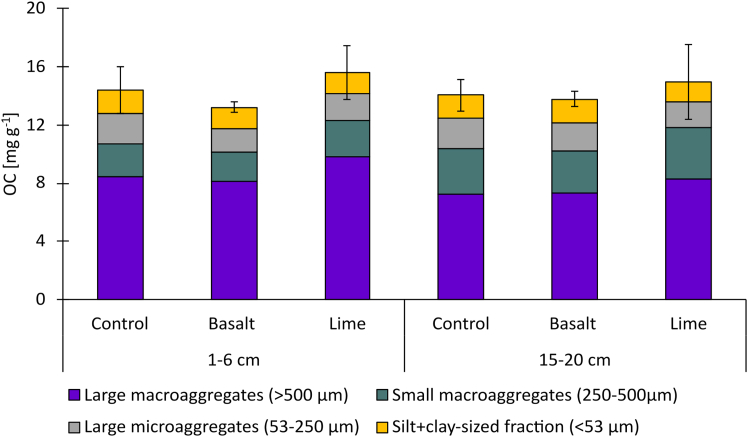
Aggregate size class contribution to the bulk soil organic carbon (OC) concentration in mg g^−1^

**Figure 3:**
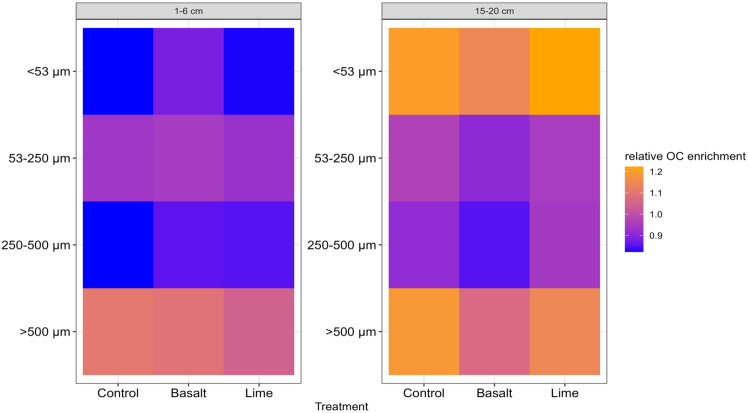
Heatmap of relative OC enrichment factors in each aggregate size class

The large macroaggregate size class (>500 μm) was the most dominant aggregate size class across all treatments (Figure 1). Among all subplots, aggregate size distributions among treatments did not differ, but there was depth stratification in all treatments, where the contribution of small macroaggregates was significantly greater in the 15–20 cm depth interval than at the surface. Aggregate size class contribution to the total SOC content did not differ between treatments, showing rock additions did not alter SOC storage in soil aggregates (Figure 2). Differences between sampling depths were visible in the small macroaggregate size class, where the relative contribution of small macroaggregates to the total SOC was significantly lower in 1–6 cm (2 mg g^−1^) than in 15–20 cm (3–4 mg g^−1^). The heatmap in Figure 3 describes relative OC enrichment (E_OC_) in each aggregate size class. SOC concentrations of all aggregate size classes and their relative E_OC_ values are provided in Table S4. The relative E_OC_ differed with soil depth (Figure 3), where the S + C-size class was more enriched in 15–20 cm (E_OC_ = 0.84–0.88) as compared to 1–6 cm (E_OC_ = 1.11–1.15) (Figure 3), where the finest fraction was less enriched.

### Changes in soil structural development and soil physicochemical properties

**Figure 4:**
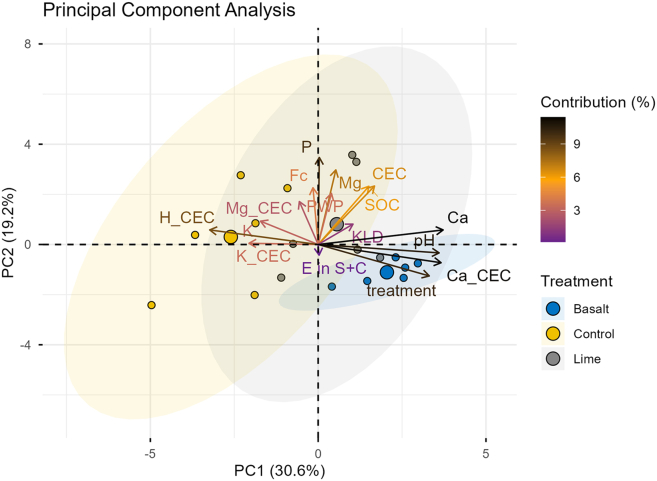
The biplot of the first two principal components, where the contribution of variables to the principal components is given in gradient colors In this figure, Fc stands for field capacity, PWP for permanent wilting point, TP, TK, TMg, and TCa stand for available nutrient concentrations (P, K, Mg, and Ca), H_CEC, K_CEC, Ca_CEC, and Mg_CEC stand for exchangeable H, K, Ca and Mg, KLD stands for Kullback-Leibler divergence, and E in S + C for relative OC enrichment in S + C-sized fraction.

**Figure 5:**
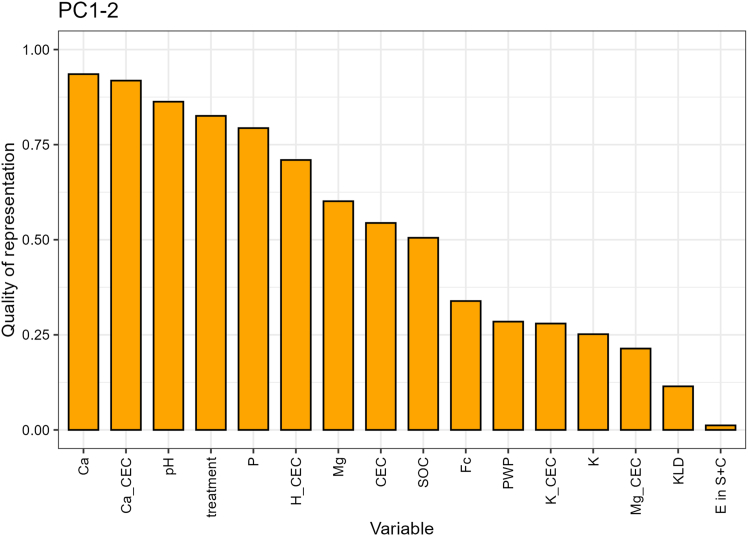
The quality of representation in the PCA

A principal component analysis (PCA) was used to summarize the multi-correlation of structural variables and their interactions between the treatments (Figure 4). The quality of representation for each variable is presented in Figure 5. Approximately 49.8% of the variation was explained by the first two principal components (Figure 4). PCA analysis showed that the pH, exchangeable Ca^2+^, available Ca concentration, treatment and exchangeable H^+^ provided the highest contribution to the PC analysis (Figure 5). The contributions of exchangeable H^+^, available K, bulk SOC concentration, available P concentration, and field capacity (Fc) were negatively correlated with the treatment.

**Figure 6:**
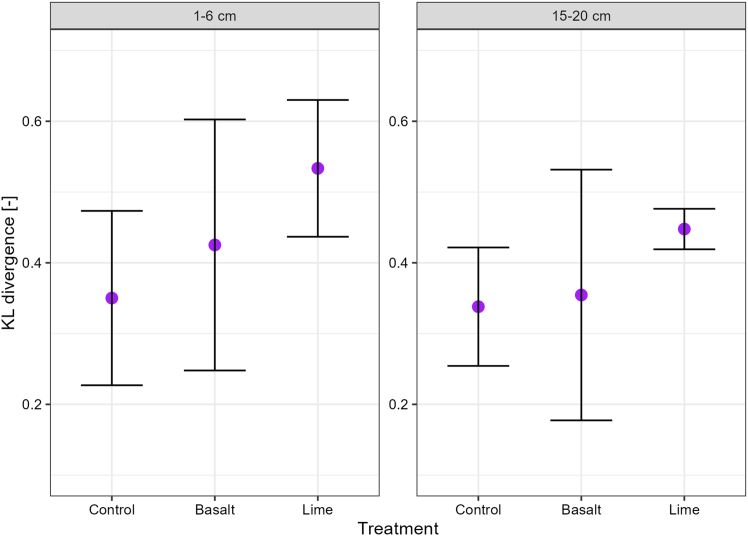
Soil structure as quantified by the Kullback-Leibler (KL) divergence KLD index is presented as mean values together with 95% confidence level.

The KL divergence (Figure 6) was significantly different between sampling depths, with higher values at 1–6 cm, suggesting that soil structure was more developed very close to the soil surface. Comparing treatments, the KL divergence was on average larger in the basalt (KLD = 0.43) and lime treatments (KLD = 0.53) as compared to the control treatment (KLD = 0.35) at the depth of 1–6 cm; however, these differences were not statistically significant. In contrast, in the 15–20 cm depth, the lime treatment had significantly higher KL divergence (KLD = 0.45) as compared to the control and basalt treatments.

## Discussion

This study at the Energy Farm investigated the long-term application of crushed basalt and its impact on chemical and physical soil properties (Figures 4 and 5). At the Energy Farm, the basalt amendment altered soil chemical quality by increasing soil pH up to 7.2, which activated soil feedback mechanisms such as shifts in nutrient availability (Table 1) and plant growth dynamics, resulting in higher crop yields.^12^ Soil pH responded to changes in soil management practices and governs subsequent biogeochemical reactions that regulate the capacity of the soil to function^34^^,^^35^^,^^36^^,^^37^ and these results highlight that pH is a primary indicator for evaluating the response of soil quality to different treatments. However, in the lime treatment, we observed that after only two years, the impact on pH had already begun to diminish as the pH had declined to 6.7 and the contribution of exchangeable H^+^ was higher than in basalt treatment (Table 1). In soils, pH fluctuates seasonally because of the production of carbonic acid, the release of H^+^ ions from OM mineralization, and nitrification of ammonium-based fertilizers, which activates buffering capacity and influences shifts in the abundance of Ca^2+^ and Mg^2+^ on exchangeable sites.^38^^,^^39^^,^^40^^,^^41^ At the Energy Farm, UAN (urea and ammonium nitrate) fertilizer was applied two out of every three years in the maize/maize/soybean rotation, which produces H^+^ ions when ammonium is nitrified. As soils acidify, bivalent cations are replaced on mineral surfaces by H^+^, as we started to observe in the lime treatment (Table 1). Despite the weakened effect of lime on soil pH, both basalt and lime treatments had a significant impact on Ca concentration and the dominance of Ca^2+^ as an exchangeable cation, all of which reflect that soil structural quality and dynamics in OM protection have probably shifted toward mechanisms that result in higher SOC accumulation.^24^

### Cation addition affects the development of soil structure

The evaluation of soil structure through KL divergence (Figure 6), which quantifies the extent of soil structural development compared to a single-grain soil structure,^42^ suggests that only the addition of lime led to a more developed soil structure. Although the KL divergence on average was the highest in the lime treatment in both sampling depths (KLD = 0.53 and KLD = 0.45, respectively), the soil structure close to the soil surface in the basalt treatment (KLD = 0.43) was more developed than in the control treatment (KLD = 0.35). Basalt weathering is a slow process by which bivalent cations are gradually released, whereas lime dissolves rapidly due to the presence of calcite, and its influence on soil structure can be quickly observed in both sampling depths.^43^^,^^44^ Vanderkloot and Ryan^45^ investigated the weathering rate of Blue Ridge and Pioneer Valley basaltic rocks, which was strongly influenced by grain size and mineralogy, with finer fractions (<45 μm) weathering at approximately double the rate of sand-sized (250–500 μm) fractions. From the mineralogy, Blue Ridge exhibited rapid Mg leaching due to chlorite dissolution, whereas Pioneer Valley basalt weathered primarily through augite and plagioclase breakdown. Considering the grain size of the basaltic rocks (Figure S3) used in this field experiment, the influence of bivalent cations on the soil structure is expected to proceed slowly. The structural responses of soil to a rock amendment can include the formation of organo-mineral or mineral-mineral interactions between clay minerals or OM and cations derived from the rock.^26^^,^^46^ In this study, we cannot identify dominant mechanism of soil structural development; however, differences in dissolution speed could explain why soil structural alterations were more favorable for the lime treatment. It has also been argued that basalt grains can affect the redistribution of soil primary particles due to the slow weathering rate of basalt. According to Rinder and Von Hagke (2021),^44^ the complete dissolution of coarse basalt grains can take several hundreds of years, which can have a negative impact on soil porosity and soil structural quality when unweathered basalt grains begin to accumulate. However, this field study revealed that basalt addition had no negative effects on these soil physical parameters after six annual applications (Table 2; Figure 5). In addition, the relationship between the high contribution of large macroaggregates and their relative OC enrichment (E_OC_ >1.0) at the surface depth suggested that soil aggregates was unaffected by accumulating unweathered basalt grains.

Soil physical parameters such as the KL divergence and soil BD showed high variability and depth stratification, which is typical of cropland soils. Conventional tillage increases the spatial heterogeneity in soil architecture by creating locally denser and looser aggregate structures and increasing the proportion of macropores throughout the plow layer.^47^^,^^48^ Over the time of several growing seasons, soil layers that are not actively used by roots will settle, resulting in higher bulk densities as seen at the lower sampling depth (Table 2). This causes soil compaction, which is characterized by a lower degree in soil structural development as compared to the surface (Figure 5). These differences in soil structural development are in line with the aggregate size distribution, in which the mass contribution of small macroaggregates and large microaggregates was higher at 15–20 cm as compared to 1–6 cm soil depth (Figure 2). Soil breaking down into finer fractions at 15–20 cm showed that the soil layer contained a high proportion of unstable aggregates, creating unfavorable environmental conditions for air and water flow, and ultimately affecting biological activity.^49^^,^^50^ These structural differences within the plow layer underline the multi-factorial dependence on the treatment, rooting depth, and tillage practices, all of which can significantly affect SOC-storage.

### Co-benefits for OC storage depend on soil management strategies

The application of basalt and lime increased available Ca concentrations, the dominance of Ca^2+^ at the exchangeable sites (Table 1), and, in the lime treatment, the development of soil structure, (Figure 6) which, in theory, should lead to an enhanced SOC stabilization in the Energy Farm field trial. The interaction between bivalent cations and clay minerals builds organo-mineral associations, which protect OC in the soil for a long time.^25^^,^^51^ However, despite the improved chemical conditions for soil structure and SOC protection, there was no impact of basalt and lime treatments on SOC concentration, neither in the bulk soil nor in aggregate size classes (Table 1; Figure 2; Table S4). However, it is important to note that the mass addition of the basalt, which is roughly 10% of the upper portion of the soil,^11^ did not lead to a significant decrease in SOC concentrations. This is in contrast to the recent study by Lei et al.,^52^ who reported a decrease in SOC concentrations in response to basalt addition after a six-month incubation experiment, likely due to increased soil pH and thus more favorable conditions for OM decomposition. However, the authors also noted that exchangeable Ca^2+^ promoted the retention and stabilization of OC, thereby counteracting SOC loss. The net change in SOC concentrations in response to basalt applications would thus be determined by the question of whether EMW or OC stabilization weighs higher. The fact that, in our study, no differences in SOC concentrations were observed after six annual applications of basalt suggests that both processes were balanced.

While the increased presence of divalent cations did not lead to any changes in SOC concentration, soil aggregation seemed to be a key mechanism in defining the soil’s ability to stabilize OM, either by physically entrapping OM in macroaggregates or chemically adsorbing decomposed OM onto mineral surfaces in the S + C-sized fraction. Our data showed that the highest proportion of SOC was stored in large macroaggregates (Figure 2) which are formed by labile OM, such as plant residues and roots.^53^^,^^54^ Labile OC mineralizes quickly in a well-aerated system,^55^ and only a small proportion will result in the mineral phase for long-term storage.^56^ In conventionally tilled soils, such accelerated OM mineralization often takes place at surface depths, which results in relative carbon depletion in the S + C-sized fraction (E_OC_ ≤ 0.88) (Figure 3). In addition, when biogeochemical reactivity is enhanced by substrate addition, such as lime, this can trigger a priming effect which intensifies the mineralization of stable OC.^57^^,^^58^^,^^59^ Nonetheless, this study provides an indication that, over a six year period, no SOC loss is observed even at high basalt application rates (Table 1).

In acidic soils SOC storage depends on the interaction between plant productivity and microbial turnover of OM that is governed by soil pH and, in strongly acidic soils, the availability of aluminum (Al).^60^^,^^61^ In case of low soil pH, exchangeable surfaces are interacting with Al, shifting SOC adsorption mechanisms toward forming resistant organo-mineral complexes with Al,^62^^,^^63^ which is important SOC stabilization mechanism for forest soils and andosols.^64^^,^^65^^,^^66^ In some agricultural soils, the continuity in acidification produces Al-toxicity (at pH < 5.5), which impedes plant growth that will have negative impact on SOC dynamics by having higher SOC losses than gains.^67^ However, in our control fields, the exchangeable Al was not present in the soil (Table 1), which counteracts possible mechanisms in C-protection that are characteristic for acidic soils. Thus, it is likely that long-term SOC storage at this site mainly depends on plant productivity and the availability of exchangeable Ca^2+^,^68^ which diminishes over time when Ca is continuously leached out.

Similar to aggregation, soil depth played an important role in carbon storage and protection, and it had a greater impact than either of the treatments (Figure 3). The organo-mineral association in the finest fraction was unaffected by rock additions, shown by similar aggregate size class distribution (Figure 1) and no OC accumulation in the finest fraction (Figures 2 and 3). There was a trend toward SOC accumulation in the finest fraction in the basalt treatment, but within the range of analytical error. Bulk SOC had a uniform distribution within the top 15 cm (Table 1) likely due to annual tillage, but observed differences in aggregate size class distribution showed that mechanisms in SOC storage differed with depth. Microbial activity and carbon turnover are depth dependent even when soils are tilled.^69^ This is evident by the OC heatmap in Figure 3, which shows that at the surface depth of 1–6 cm SOC was depleted in the S + C-sized fraction relative to the bulk soil, whereas it was enriched at 15–20 cm. The SOC in the S + C-sized fraction is protected from microbial activities by forming organo-mineral associations, hence, the deeper sampling depth showed a greater potential for long-term carbon storage, as demonstrated by the relative enrichment of OC in the S + C-sized fraction (E_OC_≥1.10). The chemical characteristics of basalt play a key role in defining whether C will be retained through binding mechanism at the molecular level or whether soil management dominates over C-stabilization mechanisms.^70^ This indicates a clear trade-off between soil management strategies and OC protection in soils (Figure 4). Nevertheless, our data show that aggregate size class fractionation is a more comprehensive measure to reflect management-induced (e.g., soil tillage, rock application) impact on SOC protection (Figures 2 and 3) than just measuring bulk SOC (Table 1), hence providing better indications for SOC storage. Although rock additions did not affect soil aggregate stability (Figure 1), the structural disturbance by tillage negatively influenced C dynamics (Figure 3), resulting in SOC-relative depletion in the S + C-sized fraction at the 1–6 cm, even when there were beneficial conditions (e.g., higher Ca concentrations) for increasing OC protection (Table 1).

### Conclusions

In a field study at the Energy Farm at University of Illinois investigated the long-term application of crushed basalt and its impact on soil chemical properties, soil structure development, and SOC concentrations. Both soil pH and the dominance of Ca^2+^ in the exchangeable cations increased in response to both treatments. However, improvements in soil chemical quality (e.g., increase in available Ca concentration) were reflected by an increase in soil structure development only in the lower topsoil of the lime treatment. Soil physical parameters, such as Kullback-Leibler divergence and soil bulk density, showed high variability and depth stratification in both treatments and control field, which is typical of conventional tillage. At 15–20 cm, soil had a higher bulk density and aggregates were broken down to finer fractions, which showed that the soil layer contained a high proportion of unstable aggregates, and thus, soil structure was less developed compared to the surface. Our data showed that depth stratification played a greater role in carbon protection than either treatment, even when there were beneficial conditions for increasing OC protection. The organo-mineral association in the finest fraction was unaffected by the basalt amendment because neither the aggregate size class distribution nor relative OC enrichment in the finest fraction differed between the control, lime, and basalt treatments. In contrast, soil depth was more important with respect to carbon storage because differences in relative OC enrichment between soil layers. This highlights that even when basalt or lime has great potential to increase OC storage because of improved soil structure and higher availability of Ca, cultivation practices such as chisel plow used in this experiment destroy soil aggregates and counteract potential benefits in OC protection. This study demonstrates that the repeated application of basalt has the potential to improve soil chemical quality, while there are no indications for changes in soil physical properties even after six years of high annual applications.

### Limitations of the study

Chemical weathering of minerals releases Ca^2+^, which has been widely agreed to have a positive effect on soil structural stability and a positive correlation with SOC concentration. Our study showed contradictory results from the theory we know, suggesting that Ca-mediated OC protection is a highly complex process, which requires further investigation. This is complicated by the fact that there are many possible mechanisms by which Ca^2+^ can interact with reactive surfaces in soil^26^; yet, there is no suitable analytical method to investigate mechanisms behind Ca-mediated interactions between OC and mineral surfaces. Recently, Shabtai et al.^71^ introduced an innovative approach to study microbe-mineral-OM interactions using ^44^Ca-labeled soils, which holds promise for advancing our understanding of calcium-mediated interactions in greater detail. A primary limitation of our study was the inability to examine how weathering influences the formation of stable organic carbon within the soil. Addressing this question would require more comprehensive laboratory analyses, such as ^13^C solid-state nuclear magnetic resonance or mesocosm experiments utilizing ^13^C-labeled OM sources, enabling the tracing of decomposition and redistribution processes of OM in basalt-amended soils.^70^

## Resource availability

### Lead contact

Further information and requests for resources should be directed to and will be fulfilled by the lead contact, Evelin Pihlap (evelin.pihlap@slu.se).

### Materials availability

This study did not generate new unique reagents.

### Data and code availability

• All data reported in this paper will be shared by the lead contact upon request, subject to their approval.
• This paper does not report original code.
• Any additional information required to reanalyze the data reported in this paper is available from the lead contact upon request.

## Acknowledgments

The authors acknowledge support from the Yale Center for Natural Carbon Capture. The authors gratefully acknowledge the two anonymous reviewers for their comments and suggestions, which improved the quality of the manuscript. We thank Vilim Filipović for his valuable advice and consultation on measurements with the HYPROP system.

## Author contributions

E.P. designed and performed the research, analyzed the data and wrote the draft of the manuscript. N.J.P. assisted with study design. N.O. and R.D.’A. aided in wet-lab chemistry analysis. D.J.B. designed the field study at the Energy Farm, and M.D.M. and I.B.K. performed the field study and aided in sampling. T.K. ran the model of Kullback-Leibler divergence. All authors contributed to discussions about the data and manuscript revision.

## Declaration of interests

N.J.P. was a co-founder of the carbon dioxide removal company Lithos but has no financial ties to the company. E.P. has affiliations with Centre of Estonian Rural Research and Knowledge (METK) and University of Tartu (UT) because of starting new position. These affiliations have not been listed on the title page of the manuscript, because METK and UT do not have any relation and contribution to the research.

## STAR★Methods

### Key resources table

**Table:** 

REAGENT or RESOURCE	SOURCE	IDENTIFIER
**Chemicals, peptides, and recombinant proteins**		

Mehlich III Extraction	A & L Great Lakes Laboratories	N/A

**Software and algorithms**		

RStudio	Open-source software	https://www.r-project.org/

**Other**		

Eltra CS analyzer	YASIC	N/A
HYPROP	METER Group	https://metergroup.com/
WP4C	METER Group	https://metergroup.com/
PARIO	METER Group	https://metergroup.com/

### Method details

#### Study site description and sampling design

The study site was located in central Illinois (40.06°N, 88.19°W) at the University of Illinois Energy Farm. In the region, the mean annual air temperature is 10.9°C and mean annual precipitation is 1051 mm.^72^ The first application of basalt at this site occurred in November 2016 using a randomized block design, which consisted of four 0.7 ha small fields and two additional large fields of 3.8 ha each.^11^^,^^12^ In this study, we sampled from small plots outlined in those studies (0.7 ha each) consisting of control (*n* = 3), basalt (*n* = 3) and lime (*n* = 3) treatment (Figure S1). Within these fields subplots (10 m × 20 m) were randomly assigned to one of the three treatments and divided into small subplots (0.1 ha each). In the basalt treated plots, two different basaltic rocks were used at different times, namely Blue Ridge basalt^73^ and Pioneer Valley basalt. The chemical composition of both basaltic rocks was similar and contained high concentrations of SiO_2_ and Al_2_O_3_, Fe_2_O_3_ and CaO (Table S2). The X-ray diffraction (XRD) analysis showed differences in mineralogical composition (Figure S2), where Blue Ridge basalt contained chlorite, actinolite, plagioclase feldspar, quartz, and epidote. Pioneer Valley basalt was dominated by chlorite, vermiculite, plagioclase feldspar, quartz, and augite. The Blue Ridge basalt contained 6% of >600 μm, 31% 250–600 μm, 27% 75–250 μm and 35% < 75 μm sized grains. (Figure S3). In comparison, the Pioneer Valley had moderately finer grain size—having 6% of >600 μm, 17% 250–600 μm, 38% 75–250 μm and 39% < 75 μm sized grains. The mass proportions of the <2 μm fraction was low in both, namely 2% for Blue Ridge and 0% for Pioneer Valley. From 2016 to 2019, the Blue Ridge basalt was applied annually (four applications) at a rate of 50 tonne ha^−1^ after crop harvest in the fall. From 2020 onward, the material was changed to Pioneer Valley basalt (two applications) with an adjusted annual application rate of 40 tonne ha^−1^. In the lime treatment plots, lime (containing calcite) was applied in April 2020 at a rate of 6.7 tonne ha^−1^ using a broadcast spreader prior to spring planting and cultivation. After each application, all plots were chisel plowed to a depth of approximately 18 cm. All fields were managed a 3-year maize–maize–soybean rotation since 2008. Before each maize planting, 28% urea and ammonium nitrate (UAN) fertilizer was applied to all plots at a rate of 168 kg ha^−1^ and then 202 kg ha^−1^ in the second maize year. No N fertilizers was applied during soybean year. Soil samples were collected in October 2022 after soybean harvest and before the next application of basalt.

The soil sampling scheme was divided into two parts, which differed in number of sampling points: a) soil chemical, and b) soil physical properties and aggregate size class fractionation. For soil chemical analysis, one sampling point was selected in each small subplot (*n* = 6) within each block (*n* = 3). (Figure S1). For the analysis of soil hydraulic properties and aggregate fractionation, one sampling point was selected for each treatment in the field. In each sampling point, undisturbed soil samples were collected at the depths of 1–6 cm and 15–20 cm using 250 cm^3^ steel soil cylinders that were pounded into the ground and removed with soil intact. These were used for measuring soil water retention and hydraulic conductivity and were stored at 4°C until analysis. Undisturbed (moist) soil samples for chemistry and soil aggregation analyses were gently broken at the plane of their weak points, sieved to <8 mm, and air-dried at 21°C.

#### Soil chemical analysis

Soil organic carbon (SOC) concentration measurements of the bulk soil and soil aggregate fractions, and TIC concentration of basaltic rocks were performed at the Yale Analytical and Stable Isotope Center (YASIC), a Yale Institute for Biospheric Studies (YIBS) research center, using a dry combustion Eltra CS analyzer. Since the control soil was carbonate free (tested with 1M HCl), and the basalt had a low TIC concentration (Blue Ridge 0.21% and Pioneer Valley Basalt 0.19%) it was assumed the total C concentration corresponds to the OC concentration. The lime treatment the total inorganic carbon content remained low (0.00–0.06 mg IC g^−1^), validating this assumption. The pH, buffer pH, available nutrient concentrations (P, K, Mg, Ca), CEC and exchangeable K, Mg, Ca, and H were analyzed by A & L Great Lakes Laboratories, Inc. (Indiana, USA). All applied methods are described in detail by NCERA-13 (2015).^74^ Soil pH was measured in a soil and water solution of 1:1 (s:w) ratio and the buffer pH was determined in a mixture of soil:water:Sikora Buffer in a ratio of 1:1:1. The nutrient content (P, K, Mg and Ca) and exchangeable cations were measured using Mehlich III Extraction (ratio 1:10) and subsequently analyzed by ICP-OES for mineral analysis.

#### Aggregate size class distribution

Aggregate fractionation was conducted to evaluate the effects of the different treatments on soil aggregate structure, aggregate physical stability, and distribution of organic matter. The aggregate size class distribution for each depth (1–6 cm and 15–20 cm) was determined using wet sieving.^52^^,^^75^ 10 g of air dried soil (<8 mm) was gently moistened on top of filter paper to avoid slaking. The pre-moistened sample was transferred to a stacked sieve tower (500 μm, 250 μm and 53 μm) to obtain four different aggregate size class fractions: large macroaggregates (>500 μm), small macroaggregates (250–500 μm), large microaggregates (53–250 μm), and the silt- and clay-sized (<53 μm) fractions (S + C-sized fraction). The soil sample was oscillated vertically at approximately 2 cm in distilled water for 2 min (30 cycles per minute), and all fractions were collected. Collected fractions were then freeze-dried and weighed to record their mass contribution. Aggregate fractionation was conducted four times for each sample to obtain sufficient sample material for further chemical analysis and sand correction. The sand content (>500 μm, 250–500 μm and 53–250 μm) in the aggregates was measured by dispersing each aggregate size class fraction with sodium hexametaphosphate (5 g L^−1^) and shaking samples for 18 h.^76^ Each fraction was then wet-sieved, dried in an oven at 60°C and its mass was recorded. We corrected for sand content by subtracting the mass contribution of sand to the aggregate fraction. We evaluated the accumulation of OC in soil aggregate size classes by calculating an relative OC enrichment (E_OC_) factor using the following equation^77^:

(Equation 1) $$E_{OC}=\frac{{OC}_{aggregate}\;\left[mg\;g^{-1}\right]}{{OC}_{bulk\;soil\;}\left[mg\;g^{-1}\right]}$$

where OC_aggregate_ is the OC concentration of the aggregate size class and OC_bulk soil_ the OC concentration in the bulk soil. An Eoc>1 indicates a relative OC enrichment and Eoc<1 a relative OC depletion of the respective aggregate size class relative to the bulk soil.

#### Soil dilution from basalt addition

During the 6 years of annual basalt applications, there was a substantial mass of basalt added on the field (Table S1). Minerals within basaltic rock have a slow weathering rate, which will have an effect on diluting soil elemental concentrations that are scarce in basalt. We estimated the basalt contribution (*B*) to the bulk soil mass and to aggregate fractions with following equation:

(Equation 2) $$B=\frac{\sum M_{basalt}}{M_{bulk\;soil}+\sum M_{basalt}}$$

where M_basalt_ is the total mass of basalt added on the field [t ha^−1^] and M_bulk soil_ is the total soil mass [t ha^−1^] for the soil depth of 18 cm, which represents the incorporation depth of basalt. The basalt contribution to the aggregate fractions was calculated based on the particle size distribution of basalt grains (Figure S3). Basalt sieving was done using Tyler sieve sizes and a 75 μm-sized sieve was the smallest size used to determine particle size distribution. Consequently, the smallest basalt fraction did not coincide exactly with the smallest aggregate size class fraction (<53 μm) and the 75 μm size limit was equalized with the 53 μm size range to calculate dilution rates. This increased the uncertainty in the dilution rate of the finest aggregates, however, dilution correction was still necessary to prevent over- and under-interpretations of elemental concentrations.

Basaltic rocks had low concentrations of OC, P and K (Table S2). Thus, the dilution in the elemental concentration of SOC, available P and K was corrected with:

(Equation 3) $${\hat{C}}_{element}=C_{element}*\left(1+B\right)$$

where ${\hat{C}}_{element}$ is the corrected elemental concentration, C_element_ is the concentration of an element and B is the basalt contribution calculated from Equation 2.

#### Soil texture

A 30 g portion of sieved (<2 mm) soil was suspended in 30% H_2_O_2_ solution and heated to 60°C. The oxidation with H_2_O_2_ was repeated until the reaction ceased. The pre-treated soil was dispersed in sodium pyrophosphate decahydrate (Na_4_P_2_O_7_ × 10 H_2_O, 40 g L^−1^) and shaken for 18 h. The silt and clay content of the suspension was measured with a PARIO automated soil particle size analyzer in the Classic mode, where the differences in suspension pressure were recorded over a measurement period of 9 h. After completing the PARIO measurement, the sand content was determined by wet sieving (500 μm, 250 μm and 53 μm) and drying sand fractions in an oven at 105°C. The data were evaluated using PARIO Control software, which calculated the primary particle size distribution based on the integral suspension pressure (ISP) method.^78^ Because of the high temperature drift, the inverse modeling of the recorded pressure data could not be computed by PARIO Control software for five measurements.

#### Soil hydraulic properties

Soil water retention and hydraulic conductivity measurements were conducted in the undisturbed soil cylinders (250 cm^3^) using an HYPROP system (METER Group, Pullman, WA, USA). First, the undisturbed soil samples were saturated with degassed water from the bottom and placed on the HYPROP device, which has two tensiometers located at cylinder depths of 1.25 and 3.75 cm. Subsequently, the soil samples were placed on a weighing scale and left drying while measuring in single-balance mode. The measurement was completed once the air-entry point of the tensiometers was reached, thus covering a range between near-saturation and a pressure head of ca. −3,000 cm. After the HYPROP measurements, water retention in the dry range was determined with the dewpoint method using a WP4C Dewpoint PotentiaMeter (METER Group, Pullman, WA, USA).^79^^,^^80^ For this, subsamples were collected from the cylinders at three different depths. Extracted subsamples were dried at 40°C and drops of water were added stepwise to create different moisture levels, followed by a 24 h equilibration period for moisture contents to stabilize.^81^ After each drop and 24 h of equilibration, a WP4C measurement was performed and the weight of the sample was recorded. After the HYPROP and WP4C measurements, all samples were dried at 105 °C for 48 h to obtain the dry mass of the soil.

The soil water retention curve (SWRC) and hydraulic conductivity curve (HCC) were derived by fitting a model to the water retention and hydraulic conductivity measurements using the software HYPROP-Fit. The bimodal Kosugi model as proposed by Romano et al.^82^ was chosen for this purpose. In this model, the soil water content (*θ*) [L^3^ L^−3^] is expressed as a function of the pressure head (*h*) [L] as follows^82^:

(Equation 4) $$\theta \left(h\right)=\theta_{r}+w\left(\theta_{s}-\theta_{r}\right)\left\{\frac{1}{2}\text{erfc}\left[\frac{\ln\left(\frac{h}{h_{m1}}\right)}{\sigma_{1}\sqrt{2}}\right]\right\}+\left(1-w\right)\left(\theta_{s}-\theta_{r}\right)\left\{\frac{1}{2}\text{erfc}\left[\frac{\ln\left(\frac{h}{h_{m2}}\right)}{\sigma_{2}\sqrt{2}}\right]\right\}$$

where *θ*_r_ denotes the residual water content [L^3^ L^−3^], *θ*_s_ the saturated water content [L^3^ L^−3^], *h*_m_ the median pore radius [L], *σ* the standard deviation [-], *w* a weighing factor [-], and erfc(.) the complementary error function. The additional subscripts “1” and “2” refer to the two pore domains. The related HCC is given as^82^:

(Equation 5) $$K\left(h\right)=K_{s}\frac{S_{e}^{\tau }}{4{\left(a+b\right)}^{2}}{\left[a\;\text{erfc}\left(\frac{\sigma_{1}}{\sqrt{2}}+{\text{erfc}}^{-1}\left({2S}_{e1}\right)\right)+b\;\text{erfc}\left(\frac{\sigma_{2}}{\sqrt{2}}+{\text{erfc}}^{-1}\left({2S}_{e2}\right)\right)\right]}^{2}$$

where

(Equation 6) $$S_{e}=\frac{{\theta -\theta }_{r}}{\theta_{s}-\theta_{r}}$$

and

(Equation 7a) $$a=\frac{w}{h_{m1}}\exp\left(\frac{\sigma_{1}^{2}}{2}\right)$$

(Equation 7b) $$b=\frac{1-w}{h_{m2}}\exp\left(\frac{\sigma_{2}^{2}}{2}\right)$$

in which *K*(*h*) denotes the hydraulic conductivity [L T^−1^], *K*_s_ the saturated hydraulic conductivity [L T^−1^], and τ is a parameter accounting for pore tortuosity and connectivity. Note that *K*_s_ was not measured but is included as a fitting parameter.

The root-mean-square error (RMSE) was used to evaluate the performance of the bimodal Kosugi model:

(Equation 8) $$RMSE=\sqrt{\frac{1}{r}\sum_{i=1}^{r}{\left[y_{i}-{\hat{y}}_{i}\right]}^{2}}$$

where *y*_*i*_and ${\hat{y}}_{i}$ are measured and model predicted water content or hydraulic conductivity, respectively.

##### Quantification of soil structure

We quantified the degree of soil structure development for all three treatments using a recently proposed index by Klöffel et al.^42^ This index requires data on particle size distribution, water retention and porosity. The index uses the concept of relative entropy, also known as the Kullback-Leibler (KL) divergence, to quantify the difference in pore-size distribution (PSD) between the structured soil and a hypothetical same soil without structural pores. The latter is referred to as the reference soil. A larger KL divergence indicates a larger difference between the two PSDs and, thereby, a larger soil structural development. The PSD of the structured soil was derived from the fitted SWRC using the simplified Young-Laplace relationship, where the pore radius (r) [cm] is a function of h^83^:

(Equation 9) $$r=\frac{0.149}{h}$$

The PSD of the reference soil was derived from the measured particle size distribution: first, the equivalent pore radii and corresponding water contents were determined using a method described by Chang et al.^84^ Subsequently, the pore radius-water content pairs were fitted using the same model as used for the structured soil (Equation 4). In doing so, *θ*_r_ was fixed to the value obtained from fitting the SWRC of the structured soil, assuming this parameter is identical for structured and reference soil. Furthermore, *θ*_s_ of the reference soil was set to 0.30.^42^^,^^85^

Having obtained the parameters of the two PSDs, the KL divergence was calculated by numerically solving the following integral^86^:

(Equation 10) $$KL\;divergence=\int_{r_{\min}}^{r_{\max}}p\left(r\right)\ln\frac{p\left(r\right)}{q\left(r\right)}dr$$

where *p*(*r*) is the PSD of the structured soil and *q*(*r*) the PSD of the reference soil. The parameters *r*_max_ and *r*_min_ refer to the maximum and minimum pore radius respectively, where the former was set to 1,490 μm and the latter to 0.1 μm respectively.^87^ The value for *r*_min_ represents the equivalent pore radius at permanent wilting point, which is assumed to be unaffected by soil structural development.^88^

### Quantification and statistical analysis

The normal distribution of the dataset was checked in RStudio (version 4.3.0) using the ShapiroWilk test, and non-parametric tests were applied to test for significance between treatments using the Kruskal-Wallis test followed by Dunn’s post hoc test.^89^^,^^90^ Differences were considered statistically significant at *p* ≤ 0.05. To determine the effect of each treatment on soil physicochemical properties, principal component analysis (PCA) was applied in RStudio using the FactoMineR package. The variables used in the PCA were normalized by standard deviation to minimize the effect of different scales and units in the dataset:

(Equation 11) $${\hat{x}}_{i}=\frac{x_{i}-mean\left(x\right)}{sd\left(x\right)}$$

where ${\hat{x}}_{i}$ denotes the normalized value, mean(x) the mean value of the variable x and sd(x) is the standard deviation of the variable x.
