# Supplementary material for: Effects of rock amendment on soil physicochemical properties and organic carbon stabilization
Source: iScience. 2025 Nov 26;28(12):114232. doi: 10.1016/j.isci.2025.114232 (PMC12756622; doi:10.1016/j.isci.2025.114232)
Supplement: Document S1. Figures S1–S5 and Tables S1–S4 [file mmc1.pdf]

iScience, Volume *28*

Supplemental information

Effects of rock amendment on soil physicochemical properties and organic carbon stabilization

Evelin Pihlap, Noemma Olagaray, Tobias Klöffel, Michael D. Masters, Rocco D'Ascanio, Ilsa B. Kantola, David J. Beerling, and Noah J. Planavsky

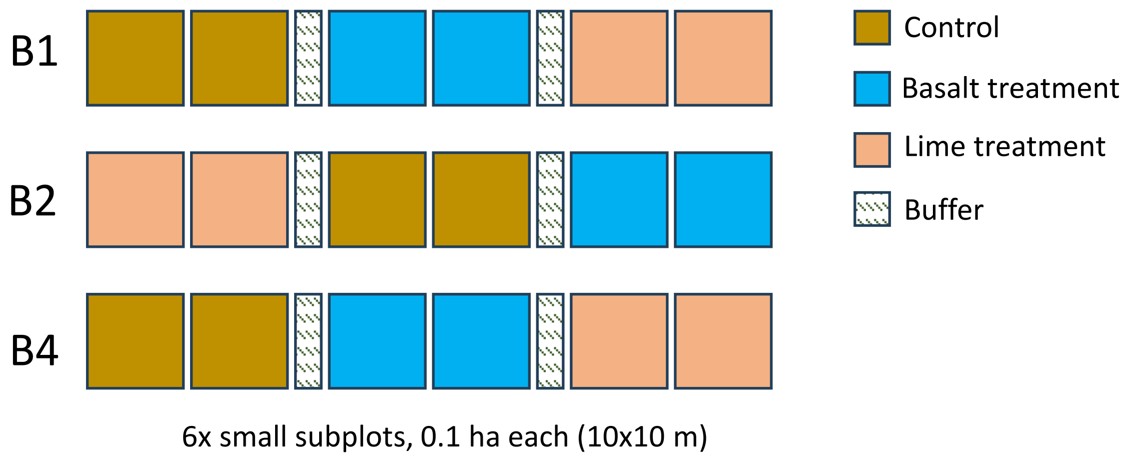

Figure S1. Block design at the experimental site at the Energy Farm.

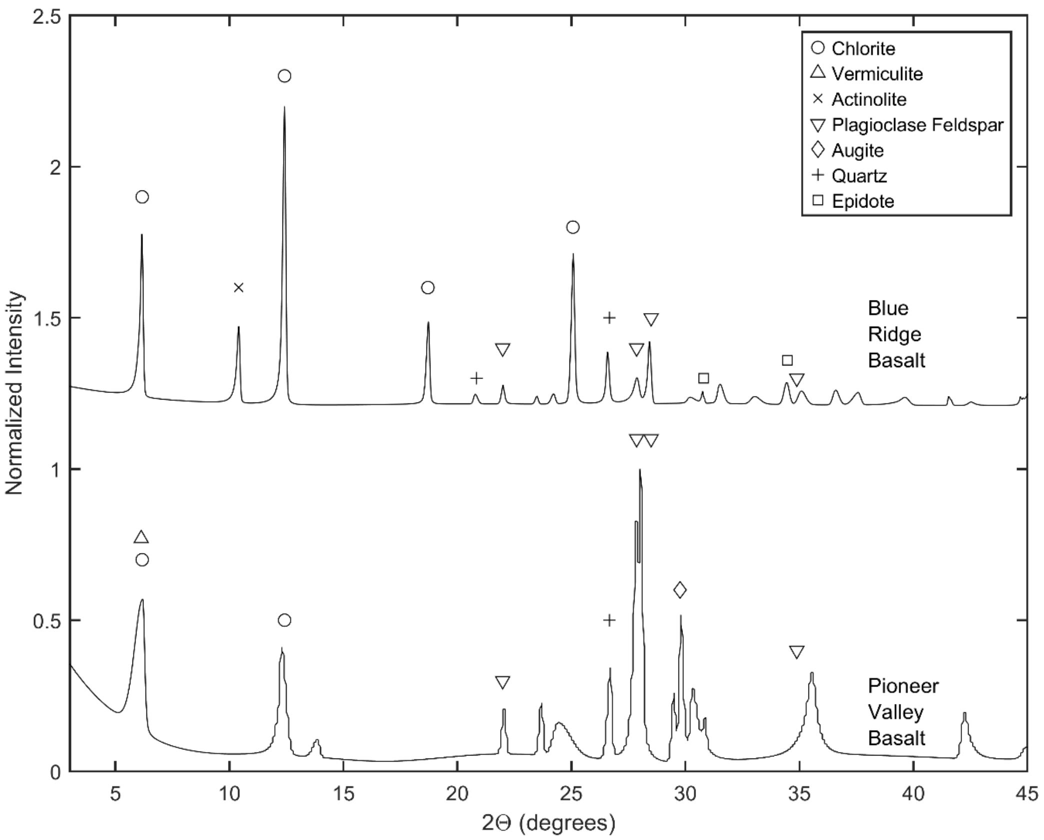

Figure S2. X-ray diffraction (XRD) data for the Blue Ridge basalt and Pioneer Valley basalt. The x-ray diffraction patterns of the basalt feedstocks were collected using a Rigaku MiniFlex benchtop powder X-ray diffraction instrument. Crushed rock samples were prepared on a glass sample slide which was loaded into the instrument for analysis. The diffraction pattern was measured over 3 – 90° 2θ at a rate of 5° per minute, and the radiation used was Cu Kα X-rays (λ = 1.5406 Å). Peak centers (2θ) in the diffraction patterns of these samples were corroborated with the diagnostic mineral peaks identified in prior work (Vanderkloot & Ryan, 2023), where the same two basalt samples were characterized using powder XRD.

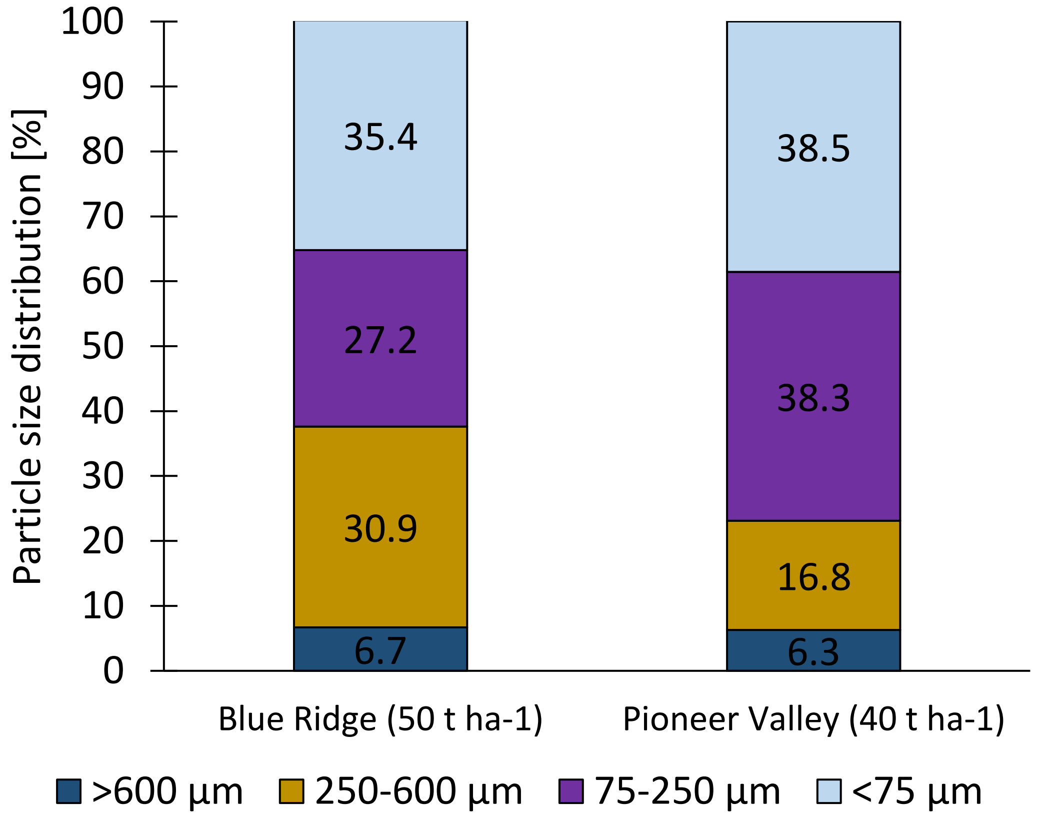

Figure S3. Particle size distribution of Blue Ridge and Pioneer Valley basalt.

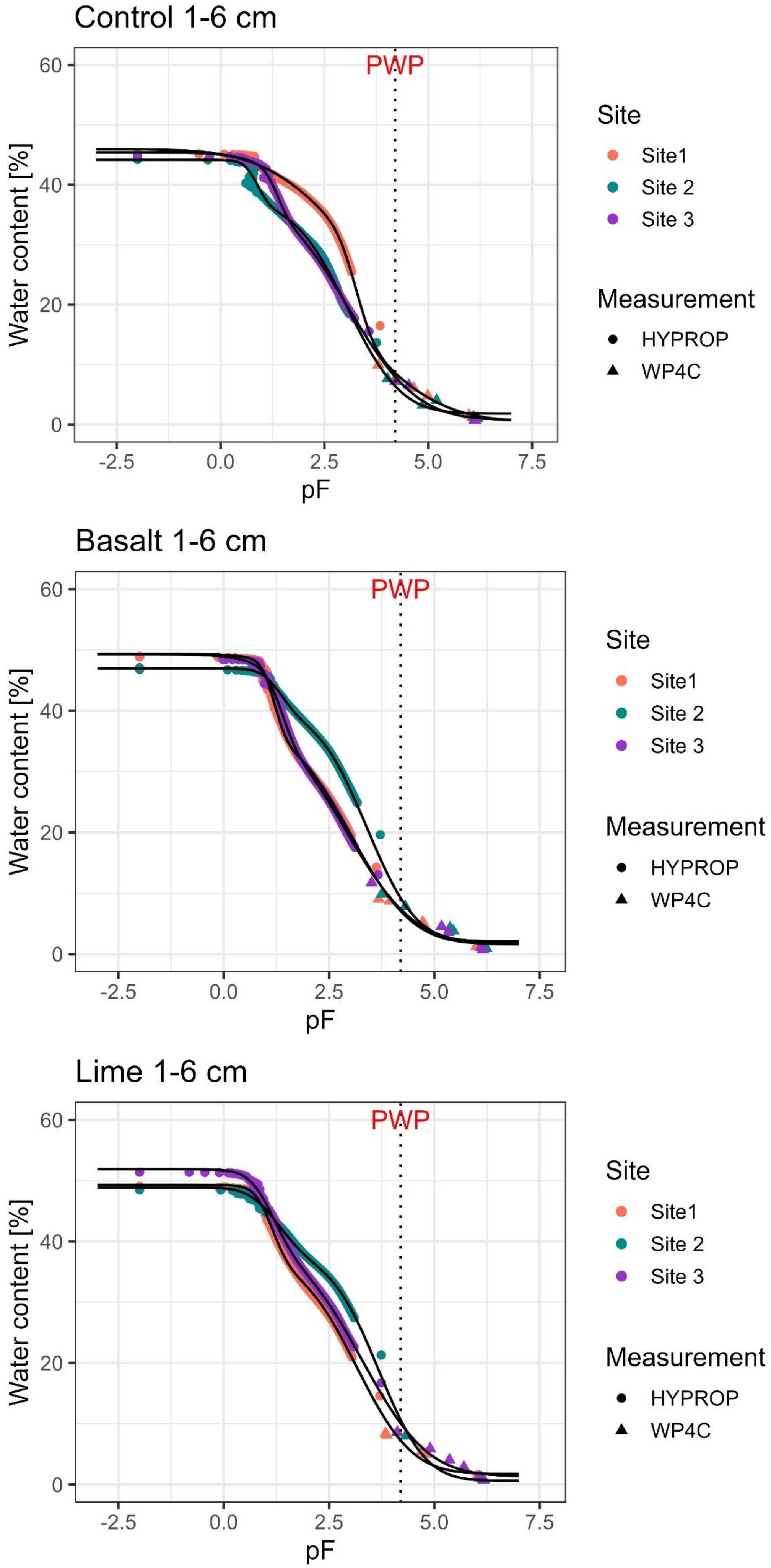

Figure S4. Water retention curve at the upper sampling depth of 1—6 cm in control, basalt and lime treatments.

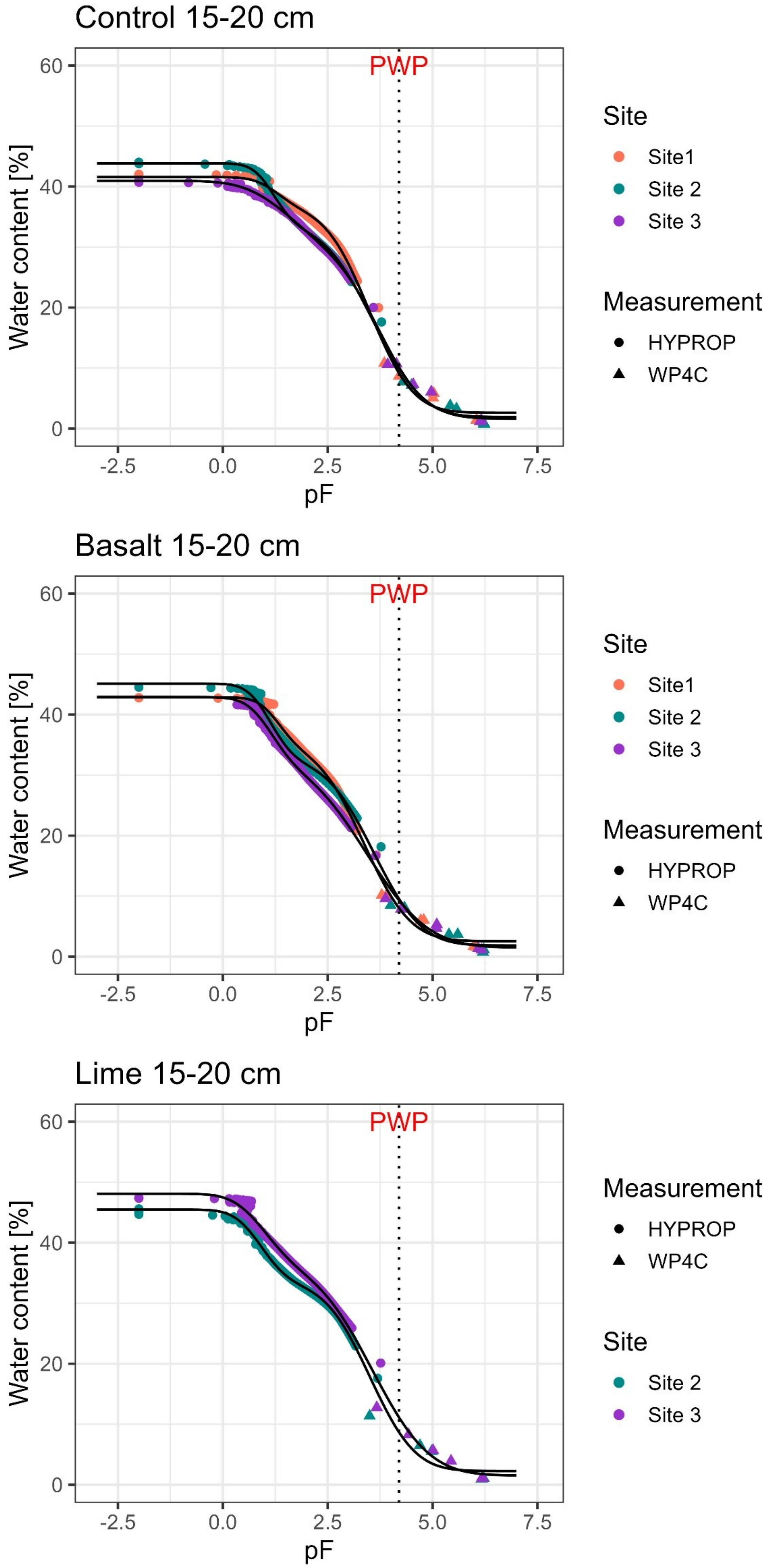

Figure S5. Water retention curve at the lower sampling depth of 15—20 cm in control, basalt and lime treatments.

Table S1. Basalt mass contribution to the bulk soil and aggregate size classes.

Year	Nº of applications	Basalt mineral	Basalt application [t ha^{-1}]	Basalt contribution to the bulk soil [%]	Basalt contribution to >630 μm fraction [%]	Basalt contribution to 250-630 μm fraction [%]	Basalt contribution to 53-250 μm fraction [%]	Basalt contribution to <53 μm fraction [%]
2015	0	-	0	0.00	0.00	0.00	0.00	0.00
2016	1	Blue Ridge	50	1.98	0.13	0.61	0.54	0.70
2017	2	Blue Ridge	50	3.89	0.26	1.20	1.06	1.38
2018	3	Blue Ridge	50	5.72	0.38	1.77	1.56	2.03
2019	4	Blue Ridge	50	7.49	0.50	2.32	2.04	2.66
2020	5	Pioneer Valley	40	8.85	0.59	2.53	2.58	3.19
2021	6	Pioneer Valley	40	10.18	0.67	2.74	3.10	3.70
2022	SAMPLING							

Table S2. Chemical composition of basalt minerals used in the Energy Farm field experiment (total amount analytes measured= 53).

	Blue Ridge [%]	Pioneer Valley [%]
SiO_2	44.2	51.6
Al_2O_3	14.8	13.6
Fe_2O_3	13.5	13.2
MnO	0.2	0.2
MgO	6.4	5.8
CaO	10.6	9.2
Na_2O	2.3	3.0
K_2O	0.3	0.9
TiO_2	2.0	1.0
P_2O_5	0.3	0.1
LOI	5.3	2.3

Table S3. Fitted soil hydraulic parameters in each study site obtained by Kosugi bimodal model.

Depth	Treatment	Site	hm1 [cm]	σ_1 [-]	θ_r [cm³ cm⁻³]	θ_s [cm³ cm⁻³]	hm2 [cm]	σ_2 [-]	w2 [-]	Ks [cm d⁻¹]	τ [-]	RMSE θ [cm³ cm⁻³]	RMSE K [cm d⁻¹]
1-6 cm	Control	Site 1	1859	0.79	0.004	0.459	1462	3.78	0.68	10000.0	-1	0.006	0.265
		Site 2	8	0.57	0.018	0.442	1134	2.35	0.83	31.8	-1	0.009	0.125
		Site 3	1142	3.02	0.008	0.454	23	0.56	0.15	924.1	0.561	0.003	0.041
	Basalt	Site 1	1052	2.65	0.016	0.493	16	0.53	0.25	243.5	0.555	0.006	0.051
		Site 2	22	0.85	0.021	0.469	2489	2.11	0.84	11.5	0.573	0.007	0.072
		Site 3	801	2.91	0.017	0.493	23	0.69	0.22	7899.3	1.862	0.004	0.070
	Lime	Site 1	14	0.75	0.018	0.493	1424	2.38	0.74	67.4	-0.373	0.006	0.044
		Site 2	4728	2.01	0.006	0.489	19	1.28	0.24	30.4	-1	0.005	0.084
		Site 3	14	0.99	0.014	0.519	1873	2.83	0.76	78.9	-1	0.004	0.061
15-20 cm	Control	Site 1	19	1.03	0.026	0.416	3065	1.90	0.87	3.8	-1	0.008	0.232
		Site 2	3872	2.18	0.016	0.438	16	0.91	0.24	9.6	-1	0.005	0.233
		Site 3	5892	1.90	0.019	0.410	39	1.97	0.30	36.2	-1	0.005	0.144
	Basalt	Site 1	21	0.91	0.026	0.429	2279	2.02	0.81	9.4	-0.8	0.007	0.077
		Site 2	14	1.02	0.018	0.451	3954	2.07	0.71	37.4	-1	0.008	0.090
		Site 3	2616	2.67	0.015	0.429	14	1.07	0.24	53.4	-0.826	0.006	0.098
	Lime	Site 2	8	1.18	0.022	0.455	3219	1.95	0.72	139.4	-1	0.008	0.096
		Site 3	9	1.36	0.015	0.481	3585	2.47	0.75	112.7	-1	0.009	0.412

Table S4. SOC concentrations and OC enrichment factors in each aggregate size class.

Depth	Treatment	SOC [mg g^{-1}] >500 µm	SOC [mg g^{-1}] 250-500 µm	SOC [mg g^{-1}] 53-250 µm	SOC [mg g^{-1}] <53 µm	E_{OC} >500 µm	E_{OC} 250-500 µm	E_{OC} 53-250 µm	E_{OC} <53 µm
1-6 cm	Control (n=3)	16.2 ±2.8	12.1 ±1.9	13.7 ±0.9	12.0 ±0.6	1.12 ±0.07	0.84 ±0.04	0.95 ±0.06	0.84 ±0.09
	Basalt (n=3)	14.6 ±0.2	11.4 ±0.7	12.7 ±0.1	11.8 ±1.9	1.09 ±0.03	0.85 ±0.05	0.95 ±0.02	0.88 ±0.15
	Lime (n=3)	16.7 ±2.1	13.7 ±2.2	14.8 ±1.7	13.2 ±1.5	1.07 ±0.01	0.88 ±0.05	0.95 ±0.03	0.85 ±0.04
15-20 cm	Control (n=3)	15.4 ±1.1	11.9 ±1.5	12.6 ±1.3	15.6 ±1.2	1.10 ±0.02	0.84 ±0.05	0.89 ±0.03	1.11 ±0.11
	Basalt (n=3)	15.3 ±0.5	12.2 ±0.6	13.0 ±0.9	16.5 ±4.3	1.07 ±0.01	0.86 ±0.06	0.91 ±0.04	1.15 ±0.26
	Lime (n=3)	16.0 ±2.2	13.2 ±2.3	13.4 ±2.5	17.2 ±3.9	1.07 ±0.05	0.88 ±0.03	0.89 ±0.02	1.15 ±0.06
